# The War against Bad Bugs: Fighting the Resistance

**DOI:** 10.3390/jcm9082563

**Published:** 2020-08-07

**Authors:** Adrian Ceccato, Antoni Torres

**Affiliations:** 1 Ciber de Enfermedades Respiratorias (Ciberes, CB06/06/0028), Institut d’Investigacions Biomèdiques August Pi i Sunyer (IDIBAPS), University of Barcelona (UB), 08036 Barcelona, Spain; aceccato@clinic.cat; 2 Intensive Care Unit, Hospital Universitari Sagrat Cor, 08029 Barcelona, Spain; 3 Department of Pneumology, Institut Clinic de Respiratori, Hospital Clínic de Barcelona, 08036 Barcelona, Spain

Multidrug-resistant (MDR) microorganisms have become a growing concern, especially in regions with high prevalence. Both the increase in antibiotic consumption and rapid spread of highly-resistant strains have contributed to a surge in MDR microorganism rates. Such a rise is significant, given that MDR organisms may also result in increased mortality and elevated healthcare costs. In fact, high mortality attributable to multidrug-resistant microorganisms for 2050 is expected in developing countries, mainly in Asia and Africa, followed by Latin America [1]. Efforts should therefore center around developing antimicrobial stewardship programs, control measures and new antibiotics.

As new antibiotics are in the process of development, old antimicrobials such as colistin or other polymyxins have been widely used instead. Although severe adverse events have been described, colistin has served as a last resort for microorganisms that are almost pandrug-resistant [2]. Yet, even in cases like that of *Klebsiella pneumoniae*, resistance mechanisms can render such types of drugs ineffective, and it is important to explore other alternatives.

*Klebsiella pneumoniae* is a Gram-negative rod that may cause respiratory, urinary, abdominal, bloodstream or disseminated disease. It is present in both community-acquired and nosocomial infections. *Klebsiella pneumoniae* can induce severe disease and is associated with several resistance mechanisms, including extended spectrum beta-lactamases, carbapenemases and resistance to second-line drugs such as colistin.

In this issue, Petrosillo et al. [3] reviewed treatment options for colistin-resistant *Klebsiella pneumoniae*. These included second-line drugs and several new promising antibiotics or combinations that had been developed in recent years, such as beta-lactamase inhibitors, cephalosporins and aminoglycosides.

One specific example is that of ceftazidime-avibactam, which has activity against carbapenemases belonging to class A and C, as well as some belonging to class D. The drug, however, does not have activity against class B carbapenemases (metallo-beta-lactamases). Ceftazidime-avibactam has been tested in nosocomial pneumonia [4], intra-abdominal infections [5] and urinary tract infections [6]. Ceftazidime avibactam has shown efficacy in 78% of MDR Enterobacteriaceae infections [7].

Another such drug being tested in in urinary, respiratory and bloodstream infections is cefiderocol. A promising tool [8] to treat MDR microorganisms, cefiderocol inhibits Gram-negative bacterial cell wall synthesis by binding to penicillin-binding proteins. It has activity against the bacteria of carbapenemase producers. The novelty of cefiderocol lies in its ability to bind free iron ions in the extracellular space, to be subsequently moved throughout the bacterial membrane by the iron transport system [9].

Finally, meropenem-vaborbactam is another drug whose results in the TANGO II study [10] were encouraging. It improved survival in a phase III study when compared with the best available therapy. High clinical recovery and lower nephrotoxicity were also observed.

While tigecycline is an available option, major concerns about high mortality and warnings issued by regulatory agencies have limited its use [11]. Tigecycline should not be administered if another therapeutic option is possible.

Additionally, Petrosillo et al. discussed non-traditional approaches such as phage therapy, antibodies and genetic editing. These types of therapies are currently in development.

Meanwhile though, until the arrival of further, new therapies, it is imperative to integrate stewardship programs to limit the overuse of already available therapies and minimize the spread of MDR microorganisms. Such an approach coupled with a sustainable plan by governments and the pharmaceutical industry to acquire breakthrough tools to fight MDR microorganisms will prove effective in this worldwide health problem. As it stands currently, pharmaceutical companies have reduced research initiatives for new antibiotics, due to lower profits. Similarly, another area worth developing is that related to diagnostic tests. Faster, improved tests could also curtail the overuse of broad-spectrum antimicrobials, and possibly mitigate associated adverse effects, too.

The war against this resistance is formidable and long; however, we are fighting the good fight, and more is yet to come.
